# Association between Asthma Control and Exposure to Greenness and Other Outdoor and Indoor Environmental Factors: A Longitudinal Study on a Cohort of Asthmatic Children

**DOI:** 10.3390/ijerph19010512

**Published:** 2022-01-04

**Authors:** Giovanna Cilluffo, Giuliana Ferrante, Salvatore Fasola, Velia Malizia, Laura Montalbano, Andrea Ranzi, Chiara Badaloni, Giovanni Viegi, Stefania La Grutta

**Affiliations:** 1 Institute for Biomedical Research and Innovation, National Research Council, 90146 Palermo, Italy; salvatore.fasola@irib.cnr.it (S.F.); velia.malizia@irib.cnr.it (V.M.); laura.montalbano@irib.cnr.it (L.M.); viegig@ifc.cnr.it (G.V.); stefania.lagrutta@irib.cnr.it (S.L.G.); 2 Department of Earth and Marine Sciences, University of Palermo, 90123 Palermo, Italy; 3 Department of Surgical Sciences, Dentistry, Gynecology and Pediatrics, Pediatric Division, University of Verona, 37134 Verona, Italy; giuliana.ferrante@univr.it; 4 Environmental Health Reference Centre, Regional Agency for Environmental Prevention of Emilia-Romagna, 41124 Modena, Italy; aranzi@arpae.it; 5 Department of Epidemiology, Lazio Regional Health Service ASL Roma 1, 00147 Rome, Italy; c.badaloni@deplazio.it; 6 Institute of Clinical Physiology (IFC), National Research Council of Italy, 56124 Pisa, Italy

**Keywords:** asthma control, children, greenness, CORINE Land Cover, land use regression, NDVI

## Abstract

Achieving and maintaining asthma control (AC) is the main goal of asthma management. Indoor and outdoor environmental factors may play an important role on AC. The aim of this longitudinal study was to evaluate the association between AC and exposure to greenness and other outdoor or indoor environmental factors in a cohort of asthmatic children. This study involved 179 asthmatic children (5–16 years). Parents were interviewed through a modified version of the SIDRIA questionnaire. AC was assessed at each visit. Exposure to greenness was measured using the normalized difference vegetation index (NDVI). A logistic regression model was applied for assessing risk factors for uncontrolled asthma (UA). Low NDVI exposure was a risk factor for UA (OR: 2.662, 95% CI (1.043–6.799)); children exposed to passive smoke during pregnancy had a higher risk of UA than those non-exposed to passive smoke during pregnancy (OR: 3.816, 95% CI (1.114–13.064)); and a unit increase in the crowding index was associated with an increased risk of UA (OR: 3.376, 95% CI (1.294–8.808)). In conclusion, the current study provided a comprehensive assessment of urban-related environmental exposures on asthma control in children, using multiple indicators of greenness and other outdoor or indoor environmental factors.

## 1. Introduction

Asthma is a major non-communicable disease affecting both children and adults [1]. Achieving and maintaining disease control is the main goal of asthma management according to GINA guidelines [2], since poor control leads to worse disease outcomes and lung function [3]. Environmental exposures may play a role in influencing asthma control and exacerbations [4,5]. To date, drawing firm conclusions about the impact of greenness on asthma in children and adolescents remains difficult [6]. Several studies investigated the role of greenness on asthma. Some cross-sectional studies have reported positive associations between greenness exposure and asthma morbidity [7,8,9] both in children and in adults [10], whilst other studies have not confirmed these findings [11,12]. Inconsistent results were also reported from other studies with longitudinal design showing both harmful [13,14] and protective [15] effects of greenness on asthma. Indeed, only one cross-sectional study assessed the potential effect of urban greenness on asthma control reporting no significant effects [16]. Asthmatic children currently exposed to indoor environmental factors, such as environmental tobacco smoke, mold and pet dander, have reported worse asthma control [17,18]. Previous cross-sectional studies have also demonstrated an association between short-term exposure to outdoor air pollutants, such as PM_2.5_, PM_10_ and SO_2_, and different asthma outcomes including asthma control [10,19]. A 15-month longitudinal study on 229 asthmatic children found an association between poor asthma control and elevated outdoor PM_2.5_ [20].

Few studies assessed the simultaneous effects of urban greenness and environmental outdoor/indoor exposures on symptoms and asthma. In particular, a recent cross-sectional study carried out in an urban area in Italy showed that multiple exposures to very low “greenness” (assessed by the normalized difference vegetation index, NDVI), “greyness” (cemented urban areas) and NO_2_ levels above the World Health Organization (WHO) recommended limits were associated with nasal, ocular, and general symptoms in 244 schoolchildren [21]. Another Italian cross-sectional study on 187 schoolchildren reported that children living in proximity to greenspaces had lower risk of having asthma in comparison with those living in less vegetated areas [22]. More recently, a cross-sectional study on the general population found that grey spaces have adverse effects on allergic status and are related to a biomarker of polycyclic aromatic hydrocarbons exposure in adulthood [23]. To date, no longitudinal study has simultaneously assessed the effect of multiple environmental factors on asthma control in children.

The aim of this longitudinal study was to evaluate the association between asthma control and exposure to greenness and other outdoor and indoor environmental factors in a cohort of asthmatic children living in the city of Palermo, Italy.

## 2. Materials and Methods

### 2.1. Study Design

Between September 2015 and December 2018, a sample of asthmatic children was consecutively recruited as a part of the CHildhood ASthma and Environment Research Study—CHASER Study, a longitudinal study carried out at the outpatient clinic of the Pediatric Allergology & Pulmonology of IRIB-CNR, Palermo, Italy. The current study involved 179 asthmatic children with repeated measurements, with a number of visits ranging between two and four in an average inter-visit time of 4.53 ± 2.86 months.

Palermo is an urban area of Southern Italy, located in the northwest of the island of Sicily, on the Gulf of Palermo in the Tyrrhenian Sea (38°06′56″ N 13°21′41″ E). There is a Mediterranean climate characterized by hot and dry summers with mild temperatures for the rest of the year. The city had 673.735 inhabitants, according to the 2017 registry office.

#### 2.1.1. Inclusion and Exclusion Criteria

The study was approved by the local Ethics Committee (Protocol N. 08/2014) and registered on ClinicalTrials.gov (ID: NCT02433275). All parents signed written informed consent. Inclusion criteria were: (1) age between 5 and 16 years; (2) diagnosis of asthma according to the Global Initiative for Asthma (GINA) recommendations (https://ginasthma.org, accessed on 1 November 2021). Exclusion criteria were: (1) immunological, metabolic, cardiac or neurological diseases; (2) major malformations of the respiratory system; (3) active smoking.

#### 2.1.2. Clinical Assessments

During the first visit, anamnestic information, including age of asthma onset, number of severe exacerbations and emergency visits in the last year, along with the assessment of personal characteristics and environmental exposures, were collected by well-trained physicians (V.M. and S.L.G.). Moreover, children underwent clinical examination and skin prick tests.

**Figure 1:**
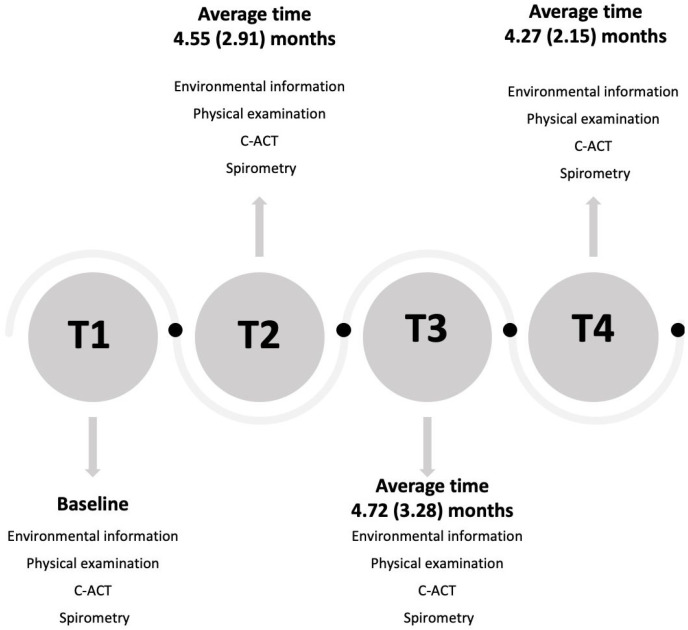
Study flowchart.

At each visit, asthma control was assessed using the Childhood Asthma Control Test (C-ACT). According to GINA guidelines, asthma severity (i.e., intermittent or persistent asthma) was retrospectively assessed based on the minimum effective level of treatment required to control symptoms and exacerbations. At each visit, parents were asked if any change in residential address or parents’ lifestyle habits, such as environmental tobacco smoke (ETS), occurred. Figure 1 illustrates the study design flowchart.

#### 2.1.3. Questionnaire

Parents were interviewed by well-trained physicians (V.M. and S.L.G.) through a modified version of the Italian Studies on Respiratory Disorders in Children and the Environment (SIDRIA) questionnaire [24]. Information about parental education, family history of asthma, physical activity and current exposure to mold (children’s bedroom), pets (cats or dogs), current ETS and maternal smoke during pregnancy (MSP) were obtained through the questionnaire. The crowding index was defined as the total number of co-residents per household, divided by the total number of rooms, excluding the kitchen and bathrooms. Current rhino-conjunctivitis was defined as a positive answer to both the questions: “Have you ever had a problem with sneezing, or runny, or blocked nose apart from common cold or flu in the last 12 months?” and “In the past 12 months, has this nose problem been accompanied by itching and/or watering eyes?”. Current eczema was defined as a positive answer to both the following questions: “In the last 12 months, have you had an itchy rush which was coming and going for at least 6 months?” and “Has this itchy rush at any time affected any of the following places: folds of elbows, behind the knees, in front of the ankles, under the buttocks, or around the neck, ears, or eyes?”. Comorbidity was defined as co-occurrence of rhino-conjunctivitis and/or eczema.

#### 2.1.4. Skin Prick Tests

Skin prick tests were performed according to EAACI recommendations with a standard panel of inhalant allergens including a positive (histamine 1%) and a negative (saline) control (ALK-Abellò, Milan, Italy). Atopy was defined as at least one positive (wheal ≥ 3 mm) skin prick test to a panel of common local aeroallergens (*dermatophagoides* mix, cat, dog, *blattella germanica*, grass mix, *parietaria judaica*, olive, alternaria). Allergens were grouped into outdoor (grass mix, parietaria judaica, olive), indoor (dermatophagoides, dog and cat dander, and blattella germanica) allergens, and spores (alternaria).

#### 2.1.5. Childhood Asthma Control Test (C-ACT) and Asthma Control Test (ACT)

The C-ACT (for children aged 5–11 years) or the ACT (for those aged ≥12 years) [25] was administered. The C-ACT includes seven items related to the previous 4 weeks, divided into two parts. The first part was filled in by the interviewed children, properly supported by their caregivers when necessary; the second part (the last three questions) was filled in by the parents/caregivers. The ACT includes five items related to the previous 4 weeks and was filled in by children. Uncontrolled asthma (UA) was defined as C-ACT or ACT total score ≤ 19; controlled asthma (C) was defined as C-ACT or ACT total score > 19.

#### 2.1.6. Spirometry Parameters

Spirometry was performed at each time visit through a hand-held turbine spirometer (Pony FX portable spirometer, Cosmed, Rome, Italy) in accordance with ATS/ERS guidelines [26]. The mean value of three valid measurements was retained. Data were expressed as a percentage of the predicted values; Z-scores were computed according to Global Lung Initiative 2012 equations that account for age, sex, race/ethnicity and height [27].

### 2.2. Environmental Exposures

#### 2.2.1. CORINE Land Cover Classes

The CORINE (coordination of information on the environment) framework is a Europe-wide satellite-based inventory of land cover developed by the European Environmental Agency in order to create a geographical information system (GIS) for providing information on the environment. The CORINE program categorized land cover into forty-four classes at a scale of 1:100,000, updated in 2006. CORINE Land Cover classes (CLC) are organized into three hierarchical levels (level 1: five categories; level 2: fifteen categories; level 3: forty-four categories) based on the unit area definition. For each home address, a class was assigned from the forty-four categories of level 3.

In our study, three classes were identified. “Artificial Surfaces” (AS) is the class in which most of the land is covered by buildings, roads and artificially surfaced areas (greyness). “Agricultural Areas” (AA) is the class in which most of the land is covered by arable land, permanent crops and heterogeneous agricultural areas. “Forest and Semi-natural Areas” (FSA) is the class mainly composed of trees, including shrub and bush, where broad-leaved species predominate. AA and FSA were aggregated in a unique category named “Natural and Semi-natural Areas” (NSA).

#### 2.2.2. Nitrogen Dioxide (NO_2_) Concentrations

For each child, exposure to NO_2_ was estimated through a land use regression (LUR) model based on the residential address, by using GIS. The LUR methodology seeks to predict pollution concentration at a given location based on surrounding land characteristics (e.g., land use, traffic intensity, proximity to emission sources, meteorology). Exposure to traffic-related air pollution was assessed for each residential address using an implemented LUR model for NO_2_ and GIS variables of the length (in meters) of high traffic roads (HTRs) (roads with >10,000 vehicles/day) within 200 m from the residential address. The European Project ESCAPE (European Study of Cohorts for Air Pollution Effects, www.escapeproject.eu, accessed on 1 November 2021) developed a standardized procedure for LUR implementation that identified common criteria for selection of sampling sites, definition of GIS predictors and development of multiple regression models [28]. In particular, linear regression models were developed using a supervised stepwise selection procedure, starting from univariate regressions of the corrected annual average concentrations with all available potential predictors, and following the procedures previously used [29]. The predictor giving the highest adjusted R^2^ was selected for inclusion in the model if the direction of the effect was consistent with the a priori definition. Then, we evaluated which of the remaining predictors further improved the adjusted R^2^ in order to select the predictor providing the highest gain in adjusted R^2^ and the expected effect direction. Subsequent variables were not selected if they changed the direction of the effect of one of the previously included variables. This process continued until there were no more variables with the expected direction of the effect adding at least 0.01 (1%) to the adjusted R^2^ of the previous model. As a final step, any variable with a *p*-value above 0.10 was removed from the LUR model. If the variance inflation factor (VIF) was >3, indicating collinearity, the variable with the highest VIF was removed and the model re-evaluated. Cook’s D statistics were used to detect influential observations. Based on the 2005 WHO Air Quality Guidelines, NO_2_ was categorized as ≥40 µg/m^3^ vs. <40 µg/m^3^.

#### 2.2.3. Normalized Difference Vegetation Index (NDVI)

NDVI is based on land surface reflectance, ranging from 0 to 1, where 0 means no vegetation and values close to 1 (i.e., 0.8–0.9) indicate the highest possible density of green leaves. In particular, the index was derived from visible red (0.63–0.69 μm) and near-Infrared (NIR, 0.76–0.86 μm) bands included in ASTER (Advanced Spaceborne Thermal Emission and Reflection Radiometer) multispectral images at 15 m × 15 m spatial resolution [30].

NDVI values were achieved for each house location involved in the study at an approximate resolution of 200 square meters, and they were categorized as ≤25th percentile vs. >25th percentile.

Since all children resided at the same address for the entire study period, CORINE Land Cover classes and NO_2_ were assessed at baseline. NDVI was evaluated at each time visit.

### 2.3. Sample Size & Statistical Analysis

Considering a small effect size (between 0.20 and 0.50) and a 5% significance level, a sample size of 103 subjects with at least two repeated measurements for subject was required in order to achieve a statistical power of 80% [31].

Data were presented as absolute and percentage frequencies or as mean and standard deviation. Categorical variables were compared using the χ^2^ test; quantitative variables were compared using the Kruskal–Wallis test to avoid distributional assumptions.

Logistic regression models, with individual-level random effects to account for repeated measurements, were applied for assessing factors associated with asthma control. Exposure variables included in the models were: NDVI ≤ 0.2176 (Y vs. N, time varying); CLC Artificial Surfaces (Y vs. N); NO_2_ ≥ 40 µg/m^3^ (Y vs. N); HTRs < 200 m (Y vs. N); ETS (Y vs. N); MSP (Y vs. N); current mold exposure (Y vs. N); current exposure to pets (Y vs. N); crowding index (continuous variable). Confounding factors included in the models were: comorbidity (Y vs. N); persistent asthma (Y vs. N, time varying); atopy (Y vs. N); parental education <8 years (Y vs. N). VIF was computed in order to assess multicollinearity. Results were reported as OR with corresponding 95% confidence intervals (95% CI). Linear mixed models, with individual-level random effects accounting for repeated measurements, were applied for assessing factors associated with spirometry parameters, expressed in Z-scores.

Two sensitivity analyses were carried out, the first one imputing missing values through a nonparametric technique based on Random Forest (implemented in the missForest R package [32]), and the second one adjusting the model also for physical activity (>3 times per week). Analyses were performed through R (R, version 4.0.2; R Foundation for Statistical Computing: Vienna, Austria, 2020); a *p*-value < 0.05 was considered statistically significant.

## 3. Results

### 3.1. Characteristics of the Study Sample

**Figure 2:**
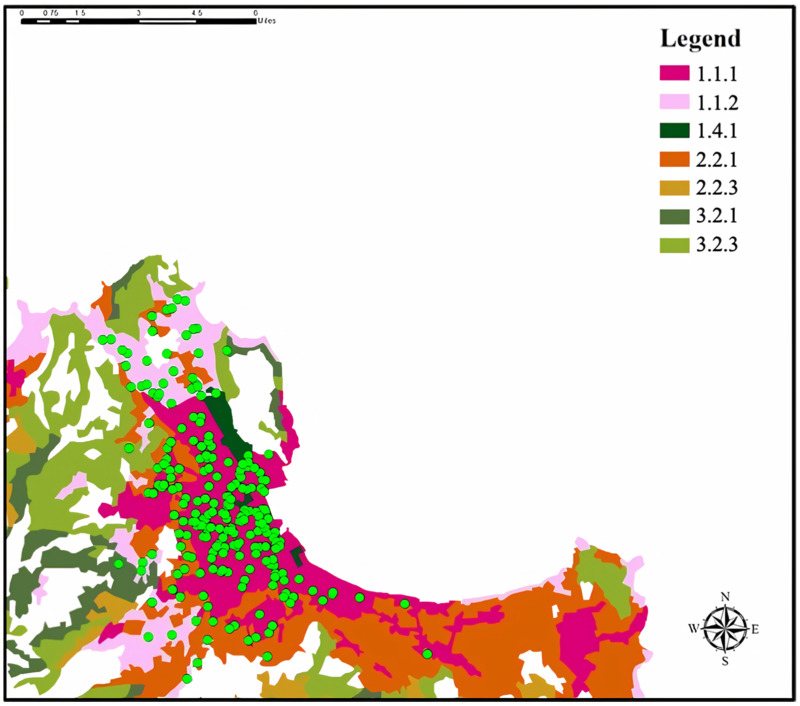
CLC category map of geo-coded children. Green points indicate the residence of each child and colored polygons identify the CORINE Land Cover categories.

Figure 2 illustrates the land cover map of the area derived from the CORINE database. Locations of children’s houses belonged to the following specific categories: 1.1.1 continuous urban fabric (n = 128); 1.1.2 discontinuous urban fabric (n = 20); 1.4.1 green urban areas (n = 5); 2.2.1 citrus grove (n = 26); 2.2.3. olive grove (n = 1); 3.2.1 natural grassland (n = 2); 3.2.3 sclerophyllous vegetation (n = 1).

Table 1 summarizes the characteristics of the study sample by asthma control status at baseline. Children in the UA group had more frequently persistent asthma, were more frequently exposed to MSP and had higher crowding index than those in the C group.

Table 2 reports spirometry parameters of the study sample by asthma control status at baseline. Children in the UA group had lower FEV_1,_ FEF_27–75%_ and FEV_1_/FVC Z-scores and percent predicted values than those in the C group.

### 3.2. Multivariable Analyses

**Figure 3:**
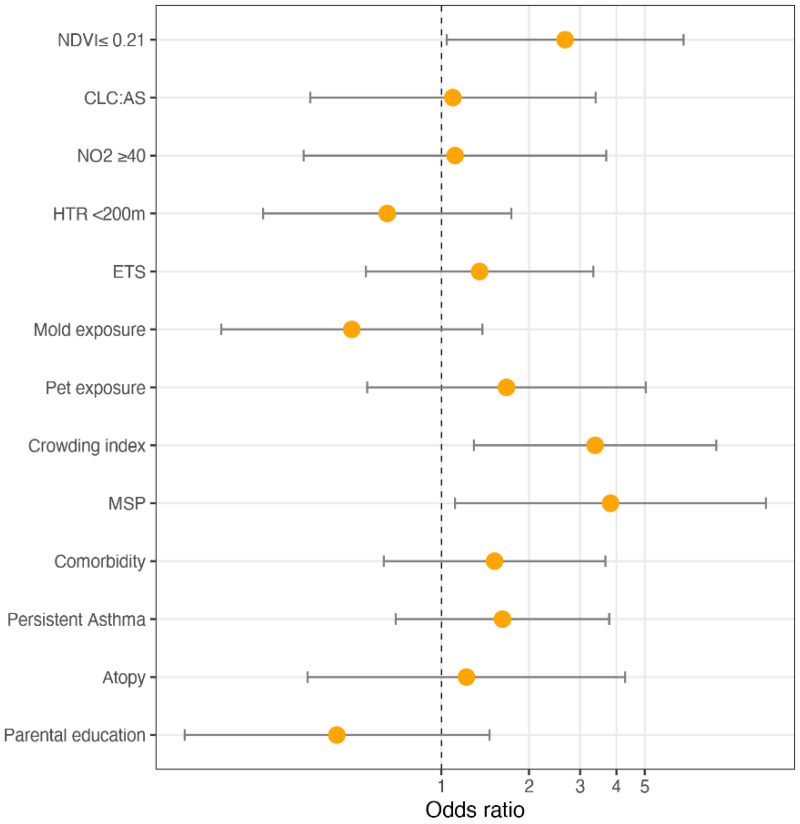
ORs and 95% CI of the mixed-effect logistic regression model for asthma control.

Figure 3 shows the ORs and 95% CI for the mixed-effect logistic regression model for asthma control. Low NDVI exposure was a risk factor for lack of control (OR: 2.662, 95% CI (1.043–6.799)); children exposed to MSP had a higher risk of UA than those non-exposed (OR: 3.816, 95% CI (1.114–13.064)); a unit increase in the crowding index was associated with an increased risk of UA (OR: 3.376, 95% CI (1.294–8.808)).

No multicollinearity concern was identified (Table 3).

With regard to spirometry parameters, no association was found among exposure to greenness and other outdoor or indoor environmental factors (data not shown).

### 3.3. Sensitivity Analyses

**Figure 4:**
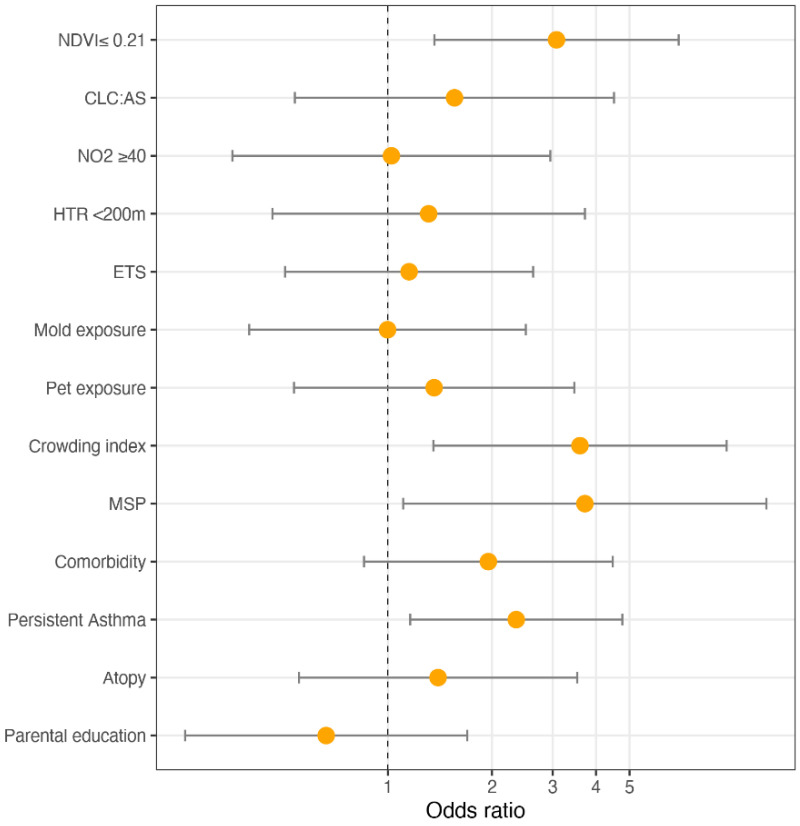
ORs and 95% CI of the mixed-effect logistic regression model for asthma control: a sensitivity analysis after missing values imputation.

Figure 4 illustrates the ORs and 95% CI from the mixed-effect logistic regression model for asthma control, after missing value imputation. Low NDVI exposure was a risk factor for lack of control (OR: 3.075, 95% CI (1.363–6.939)); children exposed to MSP had a higher risk of uncontrolled asthma than those non-exposed (OR: 3.715, 95% CI (1.108–12.454)); a unit increase in the crowding index was associated with an increased risk of UA (OR: 3.595, 95% CI (1.354–9.547)).

**Figure 5:**
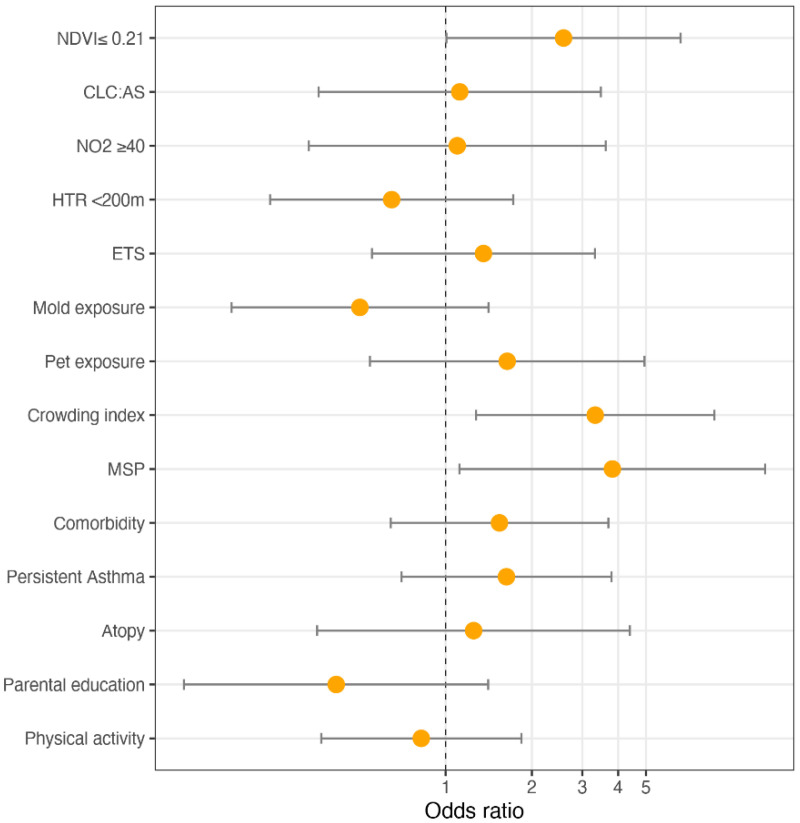
ORs and 95% CI of the mixed-effect logistic regression model for asthma control: sensitivity analysis adjusting for physical activity (>3 times per week).

Figure 5 illustrates the ORs and 95% CI from the mixed-effect logistic regression model for asthma control, after adjustment for physical activity (>3 times per week). Low NDVI exposure was a risk factor for lack of control (OR: 2.579, 95% CI (1.007–6.603)); children exposed to MSP had a higher risk of uncontrolled asthma than those non-exposed (OR: 3.819, 95% CI (1.118–13.043)); a unit increase in the crowding index was associated with an increased risk of UA (OR: 3.326, 95% CI (1.277–8.663)).

## 4. Discussion

Our longitudinal study is the first to assess the association between exposure to greenness and outdoor/indoor environmental factors with asthma control in children. Through multivariable analyses, adjusting for confounding factors, we found that a very low exposure to greenness measured by NDVI was associated with a higher risk of uncontrolled asthma: the same occurred for maternal smoke during pregnancy and higher crowding index.

In our study, uncontrolled asthma was significantly associated with a low exposure to NDVI ≤ 0.21. To date, only one study investigated the association between greenness exposure and asthma control. Differently from the current findings, Chen et al. [16] reported no effect of NDVI on asthma control in a cross-sectional study on 150 children aged 9–17 years. A possible explanation for this discordant result may be the different study design (cross-sectional vs. longitudinal). Indeed, longitudinal studies always provide larger statistical power than cross-sectional ones [33]. However, the difference cannot be ascribed only to different study design; indeed, a previous study found that, despite identical study designs and statistical modelling, greenness effects differed across two study areas in Germany [34]. Therefore, another reason of that discrepancy might be ascribed to the different a priori choice of buffer to define greenness (250 m radius vs. 7.5 m radius of our study).

There are many potential pathways through which greenness can act as a protective factor for asthma control. First, it is likely that children living near to greenspaces are less exposed to traffic pollutants. In any case, our models were adjusted for NO_2_ exposure, CLC around home address and distance from high traffic roads. Second, living in proximity to greenspaces may stimulate physical activity in children and adolescents [35,36], which has been associated with a controlled asthma status [37]. Indeed, in our study, adjusting for physical activity did not alter the observed associations.

Greenness may negatively influence asthma control through pollen exposure, as already observed [20]. It is to point out that, although it was not possible to distinguish for the specific types of vegetation in our study, our model considered atopic sensitization among confounder factors.

We found that children exposed to maternal smoke during pregnancy had a higher risk of uncontrolled asthma. Exposure to maternal smoke during pregnancy may be a risk factor for asthma development, due to the change in placental cytokine production [38], with an increased risk of atopic diseases and asthma in the offspring [39]. Moreover, maternal smoke during pregnancy may have persistent effects on lung development, lung function and respiratory health later in life [40,41]. To date, the association between asthma control and maternal smoking during pregnancy has been poorly investigated [40]. The higher risk of uncontrolled asthma linked to maternal smoke during pregnancy in our study population is in line with the findings of the CAMP study in which in utero smoke exposure appeared to blunt the beneficial effects of ICS use on airways responsiveness [42].

An increasing crowding index was associated with an enhanced risk of uncontrolled asthma in our study (OR: 3.376, 95% CI (1.294–8.808)). Residential crowding can be considered as a marker of socioeconomic status since a lower value was found to be associated with better social and financial status [43]. High crowding index leads to more stress, impairment of social relationships and sleep, and augmented risk of hypertension, respiratory illnesses and infections [44,45]. In line with our findings, crowding index has been linked to poor control in children with asthma [45,46].

No significant association was found between NO_2_ and asthma control. In contrast with our results, a negative effect of NO_2_ on childhood asthma control was found in a longitudinal study on 7211 children aged 0–14 years [47]. This discrepancy might be ascribed to the low variability of NO_2_ values in our study sample, with insufficient spatial contrasts yielding insignificant differences.

This study has several strengths. Firstly, it benefited from a simultaneous use of multiple indicators of greenness and outdoor/indoor environmental factors. Furthermore, the use of the LUR model can be considered as a strength since it has been extensively used in previous studies, showing reliable estimates [48]. Lastly, we tested the robustness of our results through sensitivity analyses.

Our study also has some limitations. Information was collected using a questionnaire, leading to a potential recall bias. However, the SIDRIA questionnaire (derived from the ISAAC one) is a standardized tool that has been widely used in previously published studies. Moreover, we did not evaluate risk factors potentially associated with urbanization, such as noise and stress, which might negatively affect asthma control in children. Finally, we took into account only NO_2_, no information about other pollutants were available.

## 5. Conclusions

In conclusion, the current study provided a comprehensive assessment of urban-related environmental exposures on asthma control in children by using multiple indicators of greenness and outdoor/indoor environmental factors. By providing new insights into the association between greenness exposure, indoor exposure and asthma control in children, the current findings may support the implementation of nature-based solutions and smoke-free policies in the urban context.

## Figures and Tables

**Table 1:** Demographic characteristics by asthma control status at baseline.

	All	C	UA	*p*-Value
n	179	129	50	
Age, years, mean (SD)	8.70 (2.64)	8.52 (2.52)	9.16 (2.90)	0.161
Gender: Female, n (%)	72 (40.22)	47 (36.43)	25 (50.00)	0.136
BMI, kg/m^2^, mean (SD)	19.36 (4.10)	19.15 (4.31)	19.90 (3.51)	0.099
Persistent asthma, n (%)	99 (55.31)	62 (48.06)	37 (74.00)	**0.003**
Median ICS dose (fluticasone propionate) μg·day^−1^	240.32 (149.02)	208.27 (132.81)	297.43 (159.58)	**0.020**
Asthma onset, years, mean (SD)	5.05 (2.59)	4.96 (2.45)	5.28 (2.32)	0.522
Severe exacerbations during the last year, mean (SD)	0.72 (1.63)	0.64 (1.31)	0.94 (2.48)	0.532
Emergency visits (at least one during last year), n (%)	26 (14.5%)	19 (14.7%)	7 (14.0%)	1.000
Atopy, n (%)	131 (76.16)	89 (72.95)	42 (84.00)	0.178
Indoor sensitization, n (%)	155 (73.81)	81 (69.23)	39 (81.25)	0.167
Outdoor sensitization, n (%)	97 (45.97)	27 (42.19)	6 (46.15)	1.000
Parental education (<8 years), n (%)	50 (28.57)	36 (28.57)	14 (28.57)	1.000
Physical activity (>3 times per week), n (%)	88 (38.60)	58 (45.31)	15 (30.00)	0.065
Comorbidity, n (%)	117 (65.73)	82 (64.06)	35 (70.00)	0.566
Environmental exposures				
Outdoor				
NDVI ≤ 0.21	39 (26.53)	28 (26.17)	11 (27.50)	1.000
CLC, Artificial Surface	148 (82.68)	106 (82.17)	42 (84.00)	0.944
NO_2_ LUR ≥ 40, µg/m^3^, n (%)	150 (83.80)	106 (82.17)	44 (88.00)	0.469
HTRs < 200 m, n (%)	145 (81.92)	104 (81.89)	41 (82.00)	1.000
Indoor				
Current ETS	62 (35.03)	43 (33.86)	19 (38.00)	0.730
Current mold exposure	38 (21.59)	22 (17.46)	16 (32.00)	0.056
Current pet exposure	38 (21.47)	28 (22.05)	10 (20.00)	1.000
Crowding index	1.10 (0.47)	1.05 (0.44)	1.25 (0.50)	**0.020**
MSP	16 (9.04)	7 (5.51)	9 (18.00)	**0.020**

UA: ACT/C-ACT ≤ 19, C: ACT/C-ACT > 19, NDVI: normalized difference vegetation index; CLC: CORINE Land Cover; HTRs: high traffic roads; ETS: environmental tobacco smoke; MSP: maternal smoke during pregnancy. *p*-values in bold are statistically significant.

**Table 2:** Spirometry parameters by asthma control.

	All	C	UA	*p*-Value
n	179	129	50	
FEV_1_, L	1.76 (0.61)	1.74 (0.60)	1.77 (0.63)	0.787
Z-score	−0.35 (1.26)	−0.23 (1.02)	−0.43 (1.79)	**0.044**
% pred	95.74 (14.71)	97.22 (12.27)	94.63 (20.09)	**0.042**
FVC, L	2.08 (0.76)	2.03 (0.72)	2.16 (0.78)	0.276
Z-score	−0.05 (1.19)	−0.03 (0.96)	0.16 (1.72)	0.921
% pred	99.54 (14.23)	99.70 (11.69)	102.01 (20.38)	0.930
FEV_1_/FVC	0.85 (0.07)	0.86 (0.06)	0.83 (0.09)	**0.032**
Z-score	−0.51 (1.01)	−0.37 (0.91)	−0.91 (1.07)	**0.014**
% pred	95.78 (7.99)	96.97 (6.43)	92.64 (9.36)	**0.015**
FEF_25–75%_, L/s	1.95 (0.84)	1.92 (0.73)	1.90 (0.90)	0.345
Z-score	−0.67 (1.29)	−0.61 (0.90)	−0.88 (1.73)	**0.022**
% pred	86.55 (35.21)	86.84 (20.14)	82.55 (40.42)	**0.023**

FEV_1_: forced expiratory volume in 1 s; FVC: forced vital capacity; FEF_25–75%_: forced mid-expiratory flow. *p*-values in bold are statistically significant.

**Table 3:** Variance Inflation Factor.

	VIF
NDVI ≤ 0.21	1.138
CLC, AS	1.318
NO_2_ LUR ≥ 40 µg/m^3^	1.340
HTR < 200 m	1.214
Current ETS	1.338
Current mold exposure	1.283
Current pet exposure	1.313
Crowding index	1.975
MSP	1.222
Comorbidity	1.164
Persistent asthma	1.137
Atopy	1.144
Parental education	1.794

NDVI: normalized difference vegetation index; CLC: CORINE Land Cover; AS: Artificial Surface; HTRs: high traffic roads; ETS: environmental tobacco smoke; MSP: maternal smoke during pregnancy.

## Data Availability

The data that support the findings of this study are available from the corresponding author, upon reasonable request.
